# An unusual case of orbito-frontal rod fence stab injury with a good outcome

**DOI:** 10.1186/1471-2482-13-31

**Published:** 2013-08-13

**Authors:** Massimo Miscusi, Paolo Arangio, Luca De Martino, Fabio De-Giorgio, Piero Cascone, Antonino Raco

**Affiliations:** 1 Department of Medical and Surgical Sciences and Biotechnologies, Rome, Italy; 2 NESMOS Department, Rome, Italy; 3 Institute of Legal Medicine, Catholic University of the Sacred Heart, 00168 Rome, Italy; 4 Department of Odontostomatologic and Maxillo-facial Sciences, Sapienza University of Rome, Rome, Italy

**Keywords:** Rod, Penetrating head injury, Orbital trauma, Cranial reconstruction

## Abstract

**Background:**

High-energy non-missile penetrating injuries (stab injuries) account for a small percentage of penetrating head injuries and they present a series of special features.

**Case presentation:**

A 35-year-old man suffered orbito-frontal? and trans-cranial injuries after falling five meters from a terrace onto a rod iron fence. The removal of the metal rod was performed outside the operating room. The orbital roof was exposed and repaired through a bifrontal craniotomy and the frontal sinuses were cranialised. The orbital floor and zygoma were plated with micro-screws.

**Conclusion:**

The patient recovered without significant complications, apart from a slight paresis of the right superior rectus; the ocular globe remained intact.

The positive outcome obtained in this very challenging case is attributable to the competency of the Neurotrauma Unit and to the use of a synergistic approach which involved the contribution of neurosurgeons, maxillo-facial surgeons, radiologists and anaesthesiologists.

## Background

High-energy non-missile penetrating injuries, also known as stab injuries, account for a small percentage of penetrating head injuries. They may be caused by knives, nails, spikes, forks, scissors, and other assorted objects. Penetration most commonly occurs through the thin bones of the skull, especially through the orbital surfaces and in the squamous portion of the temporal bone. The mechanisms of neuronal and vascular injury caused by cranial stab wounds may differ from those caused by other types of head trauma. Unlike missile injuries, no concentric zone of coagulative necrosis caused by dissipated energy is present. Unlike motor vehicle accidents, no diffuse shearing injury to the brain occurs. Unless an associated hematoma or infarct is present, cerebral damage caused by stabbing is largely restricted to the wound tract. Sometimes, a narrow elongated defect, or so-called slot fracture, is produced and diagnosed when identified [1]. However, in some cases in which skull penetration is proven, no radiological abnormality can be identified. In a series of stab wounds, de Villiers reported a mortality of 17%, mostly related to vascular injury and massive intracerebral hematomas [2]. Infections can easily complicate penetrating craniocerebral injuries and patients can develop meningitis, epidural abscess, subdural empyemas, or brain abscess. Therefore, prevention and proper management of infectious complications can lead to improved outcomes in these patients.

## Case presentation

**Figure 1:**
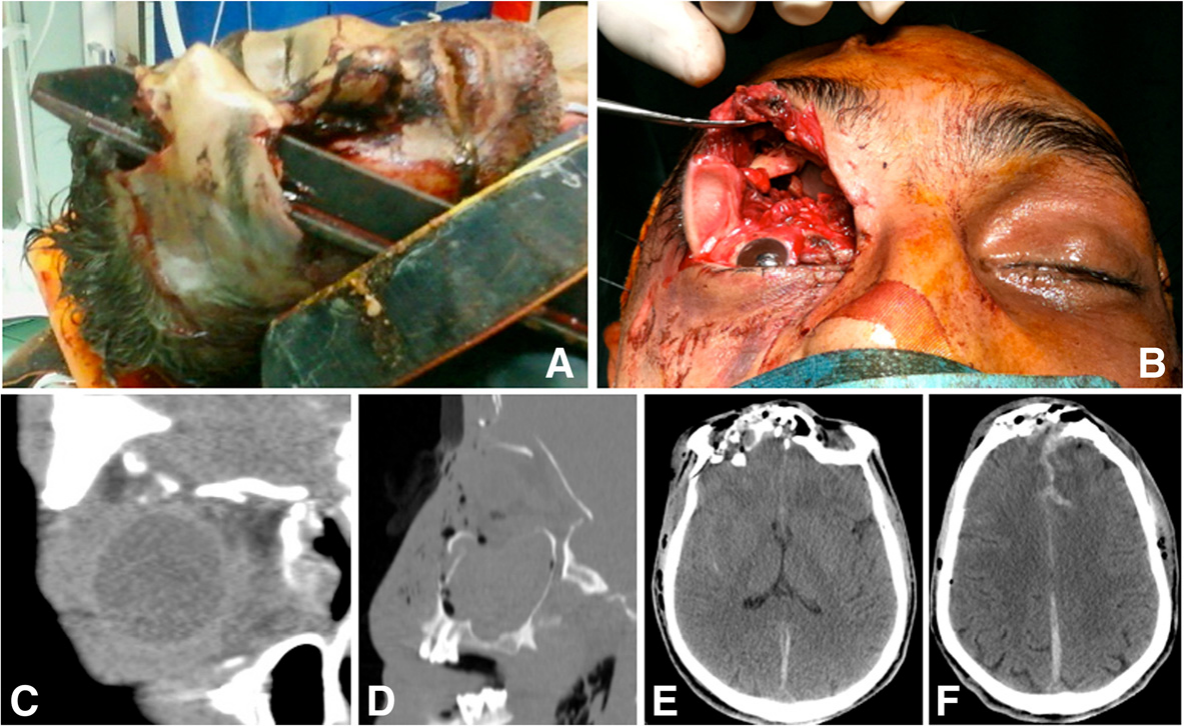
**Patient at emergency department. A: **Patient at our Emergency Department with the metal rod still embedded into the right orbit and skull. **B:** Inspection of the right orbit, showing a complete destruction of orbit roof with the ocular globe pushed backwards and downwards. **C** and **D:** CT coronal and sagittal scan reconstructions showing the fracture of both orbit floor and roof. **E** and **F:** CT axial scans at admittance, demonstrating the fracture of frontal bone, with opening of frontal sinus, and signs of subarachnoid hemorrhage in the inter hemispheric cistern.

We present the case of a 35-year-old man who suffered a high-energy non-missile orbito-frontal? penetrating brain injury after falling five meters from a terrace onto a rod iron fence. The patient was transported to our Emergency Department with the rod still embedded into his right orbit and skull (Figure 1A).

The GCS score on admittance was 6. The rod was removed in the Emergency Room without any radiological control; a consequent CT scan revealed that the injury involved the right orbit floor, roof and zygoma, both the frontal sinus and the frontal lobes and had caused a frontal post-traumatic acute subdural haemorrhage. The right ocular globe was intact because the “burst” fracture of the orbital compartment (a comminute fracture of the floor, roof, lateral and medial orbital walls) allowed the ocular globe to undergo a downward displacement without being smashed against an osseous wall (Figure 1B-E). Cranial and maxillo-facial CT scans excluded signs of vascular damage.

**Figure 2:**
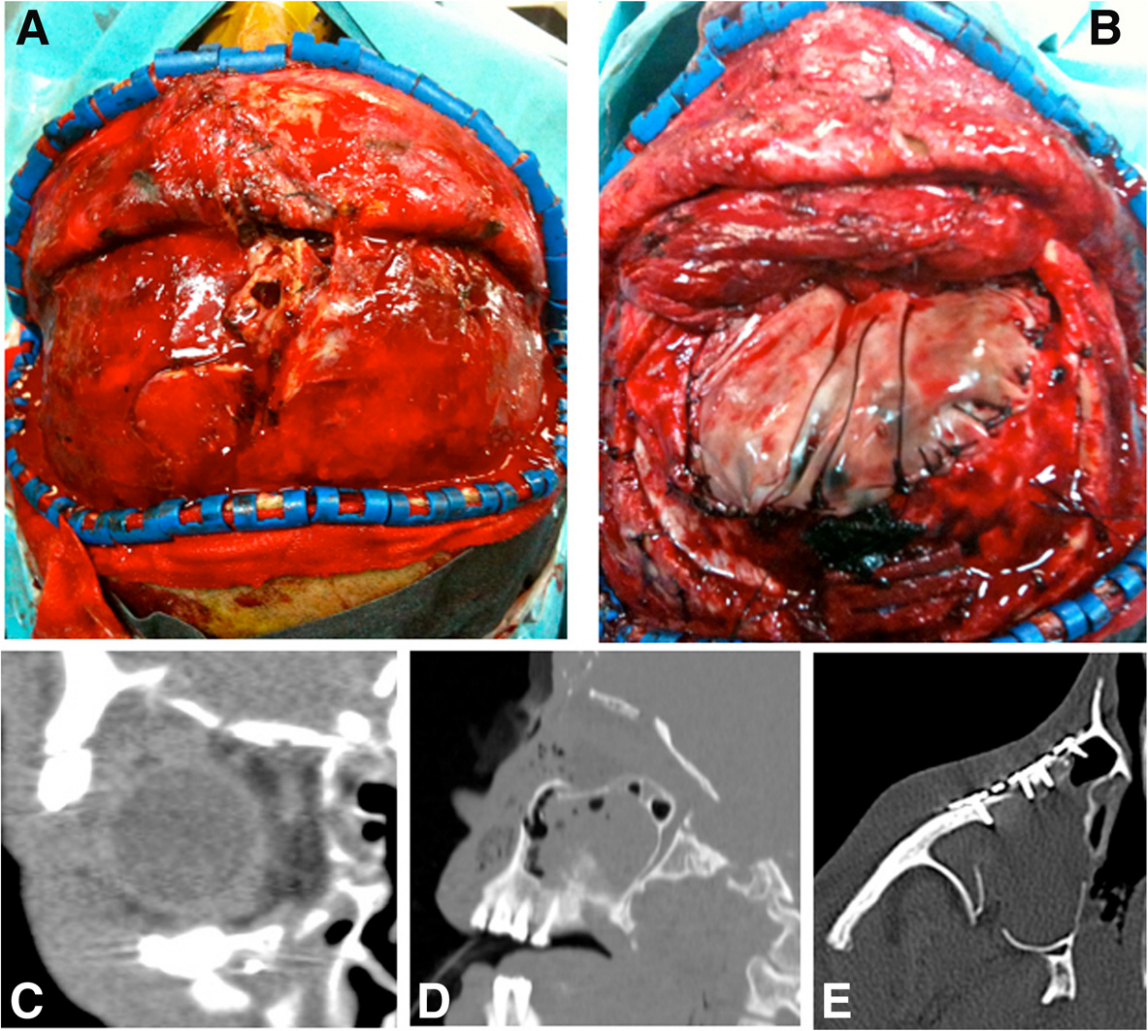
**Skull and maxilla reconstruction: surgical and radiological view. A:** Intraoperative view at the opening, with fracture of frontal bone and necrotic brain through the bone gap. **B:** Intraoperative view after duroplasty and cranialisation of the frontal sinus by galeal flap. **C, D** and **E:** CT scan, coronal, sagittal and axial scans, demonstrating the reconstruction of the orbit floor and roof and zygoma.

According to our imaging and treatment protocols, an angiogram was not performed because, considering the location and trajectory of the foreign body, there was no evidence of possible vascular injury. The patient immediately underwent surgery to evacuate the subdural haemorrhage and to repair both cranial and facial defects. Neurosurgeons and maxillo-facial surgeons participated simultaneously in the procedure. A bifrontal craniotomy was performed through a bicoronal flap. Intracranially, the dura mater and both frontal lobes with the first third of the superior longitudinal sinus were lacerated (Figure 2A). Haemorrhagic and necrotic tissue was debrided and the superior longitudinal sinus was clipped without difficulty. Rust was found inside the parenchyma and was removed together with (through??) a meticulous irrigation of the operative field. The frontal sinuses were cranialised, the mucosa was removed from the nasofrontal ducts. After cranialization, the nasofrontal ducts were obliterated using the temporalis muscle flap, fibrin sealant was applied over the ducts, and the pericranial flap appropriately folded in order to obliterate the dead space of the sinus. A large-scale duroplasty was performed with a bovine pericardial patch (Figure 2B). A right eyelid incision was performed to expose and reduce margin fractures using a titanium plate and screws (Figure 2C-E). A collagen membrane was used for orbital floor and roof reconstruction.

The patient was then moved to the intensive care unit and his neurological status showed a progressive and constant improvement; after fifteen days, sedation was stopped and the patient was discharged from the intensive care unit.

A large spectrum antimicrobial therapy (gram+, gram-, anaerobics and fungi) was administered continuously for one month because of the high risk of intracranial septic complications, which at the end did not occur.

**Figure 3:**
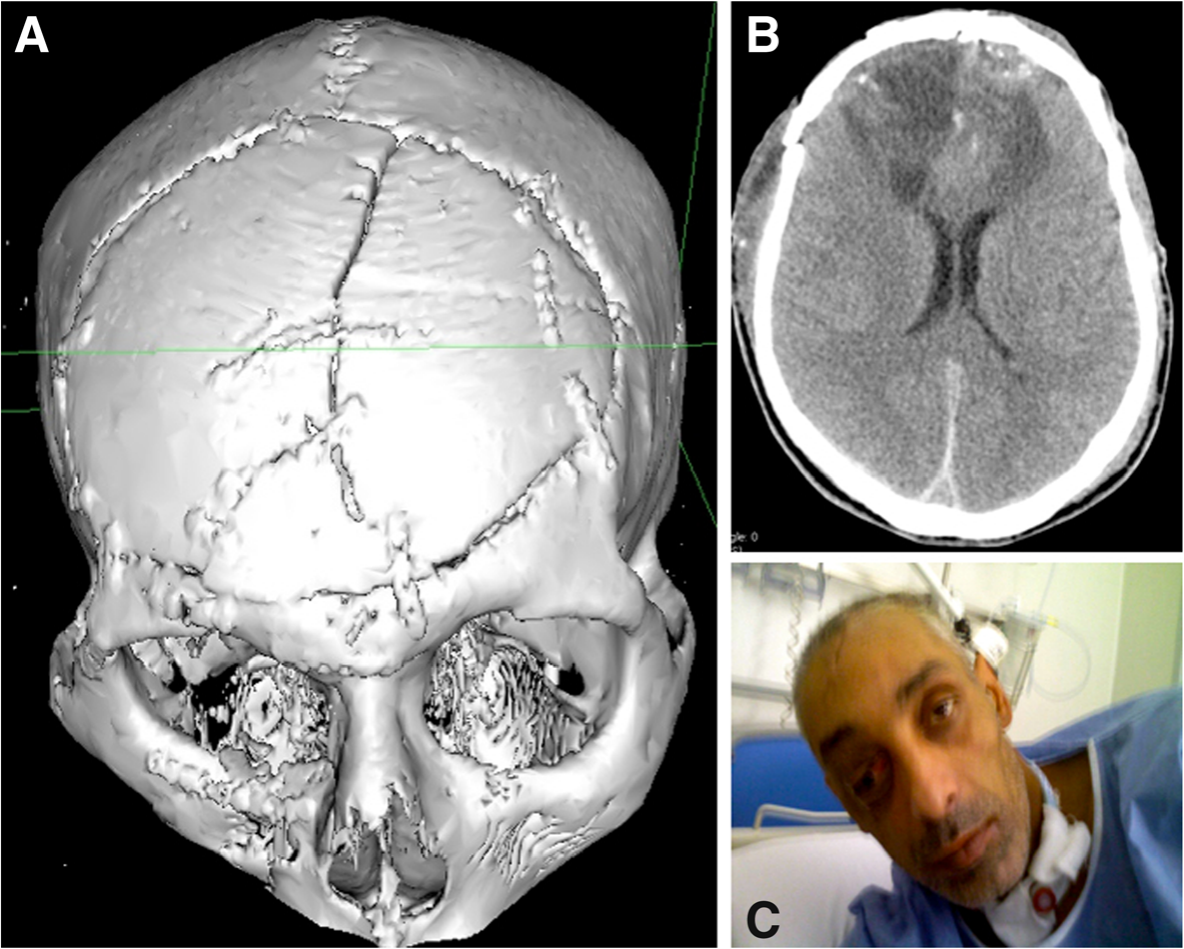
**Clinical and radiological outcome. A: **3D CT scan view showing the final reconstruction of the fractured frontal bone. **B:** CT scan performed one month after injury, demonstrating the hypodensity of both frontal lobes as chronic consequences of penetrating injury. **C:** Patient at discharge with paresis of right superior rectus muscle and ptosis.

A CT scan, performed one month after injury, showed a good reconstruction of the cranial vault, of the orbital floor and of the roof and demonstrated the chronic evolution of the frontal lobe injury (Figure 3A-B).

The patient was discharged to a rehabilitation unit in good general condition (GCS 15), without focal neurological signs. The right ocular globe was completely undamaged and visual acuity remained intact; however, a mild paresis of right superior rectus muscle and a slight ptosis were evident (Figure 3C). The patient’s neurological status and visual acuity were unchanged at the eight-month follow-up and he went back to being a street musician. He refused to undergo neuro-psychological testing to assess the frontal abilities.

## Conclusions

Penetrating stab intracranial injuries caused by metallic foreign bodies are very rare among the civilian population. Only a few cases have been reported in the literature in the last two decades, but medical reports of stab wounds of the brain date from as early as 1806 [3-10]. Penfield described the pathological features of experimental stab wounds with cannulas [11]. The literature provides a long list of objects known to have penetrated the brain, which include knives, chopsticks, pitchforks, crochet hooks, knitting needles, brooch pins, umbrella tips, crowbars and iron rods, car antennas and scissors [12-20].

To our knowledge, this is the first report of a high-energy penetrating brain injury caused by such an object.

Penetrating craniocerebral stab injuries are more amenable to treatment than missile injuries. A stab wound creates a narrow hemorrhagic infarction which is mainly restricted to the wound tract. Concentric zones of coagulative necrosis do not result from stab wounds as they do from explosives and from the cavitating forces of missiles. Similarly, contre-coup injuries rarely occur, if at all, from stab wounds [21]. Unless the stabbing instrument is swept across the brain before withdrawal, the resultant lesion is usually focal. Therefore, when there are no direct injuries to the brain stem or direct lacerations of a major vessel, the prognosis for recovery in such type of cranial injury may be good.

Frontal stabs are accompanied by the least morbidity and mortality, while penetrating orbito-cranial stab injuries characteristically cause considerable morbidity and mortality by direct damage to brain structures, vascular compromise, or infection [22].

Vascular compromise may be due to direct vascular trauma of the internal carotid artery both in its intracavernous and paraclinoid segment, to distortion of the vessel due to local effects such as haematoma formation or oedema, or vasospasm [23].

Non-contrast cranial and maxillofacial CT scan, which is the best imaging modality for this type of trauma, can help analyze the trajectory of the foreign body and observe whether the object went through any areas of major vascular significance, including the ICA (especially within the cavernous sinus) and the anterior and middle cerebral arteries. In case of suspicion for vascular injury, an angiography should also be performed to evaluate for traumatic aneurysm, which can develop soon after a penetrating injury [17,18].

In our case, in spite of the fact that the removal of the metal rod from the orbit and skull was performed outside the operating room without any radiological control, the early surgical treatment proved to be effective. Patients in whom the penetrating object is left in place until surgical removal have a significantly lower mortality than those in whom the objects are inserted and then removed (26% versus 11%, respectively) [24].

Blind removal of the intracranial object may cause neurological and vascular injuries if the trajectory is close to major vessels or to important neural structures. Therefore, an adequate surgical access allowing intracranial direct visualisation of the object should be performed before its withdrawal [15]. Another possibility is to remove the foreign body under CT control to enable prompt detection and management of any complications; in this case, both operating theatre and angiography should be ready [17]. In patients in whom there is no CT evidence of intracranial haemorrhage or possible damage to vascular structures, the penetrating object can be directly removed before surgical repair under general anaesthesia [18]. In our patient, even if the trajectory of the rod excluded a major vascular injury, the decision to withdraw the object blindly was made hastily because it could have been fatal for the optic nerve, compromising the visual acuity.

Surgical reparation of orbito-cranial and facial injuries was performed in a single surgical procedure by a multidisciplinary team, which included neurosurgeons and maxillo-facial surgeons.

Despite the high risk of cerebral infection due to polimicrobial contamination of intracranial compartment, our patient, who received a large spectrum antibiotics therapy, did not present any sign of meningo-encephalitis. Administration of anti-tetanus serum and antibiotics, and wound debridment by oxygen peroxide may minimise infectious complications [22].

According to the literature, antibiotic therapy should be initiated on admission and, although there are no data to support continuation of antibiotics after surgical removal, we strongly suggest to continue their administration for at least three weeks and in any case not less than one week [17,18].

Furthermore, surgical interventions can achieve also a good aesthetic result, with correction of facial and cranial vault deformities. The patient presented only a slight paresis of the right superior rectus muscle due to its lacerations secondary to the passage of the rod fence through the thin orbital roof. No damage to neural structures and no sign of internal carotid involvement were demonstrated.

In conclusion, this is a unique case of penetrating orbito-cranial stab injury. Despite the removal of the rod fence from the intracranial compartment through the orbit, we obtained a very good result. The positive outcome obtained in this very challenging case is attributable to the competency of the Neurotrauma Unit and to the use of a synergistic approach. This was possible because in the Unit there is a consolidated tradition of fruitful cooperation between neurosurgeons, maxillo-facial surgeons, radiologists and anaesthesiologists.

## Consent

Written informed consent was obtained from the patient for publication of this Case report and any accompanying images. A copy of the written consent is available for review by the Series Editor of this journal.

## Competing interests

Authors declare that they have no competing interests.

## Authors’ contributions

MM conceived of the study and wrote the paper. PA performed the review of the literature and helped to write the paper. LDM helped to write the paper and to design the manuscript. FDG helped to review the literature and to draft the manuscript. PC helped to write the paper and to design the manuscript. AR participated in the coordination of the study. All authors read and approved the final manuscript.

## Pre-publication history

The pre-publication history for this paper can be accessed here:

http://www.biomedcentral.com/1471-2482/13/31/prepub

## References

[B1] Bozzeto-Ambrosi P Costa LF Azevedo-Filho H Penetrating screwdriver wound to the head Arq Neuropsiquiatr 2008 93 95 18392426 10.1590/s0004-282x2008000100024

[B2] De Villiers JC Sixteen cases of transorbital stab wounds of the head J Neurol Neurosurg Psychiatry 1975 38 8 822 1185203 10.1136/jnnp.38.8.822-a PMC492081

[B3] Di Roio C Jourdan C Mottolese C Convert J Artru F Craniocerebral injury resulting from transorbital stick penetration in children Childs Nerv Syst 2000 16 8 503 506 10.1007/s003810000291 11007502

[B4] Domenicucci M Qasho R Ciappetta P Vangelista T Delfini R Surgical treatment of penetrating orbito-cranial injuries. Case report J Neurosurg Sci 1999 43 3 229 234 10817393

[B5] Karim T Topno M An unusual case of penetrating head injury in a child J Emerg Trauma Shock 2010 3 2 197 198 10.4103/0974-2700.62113 20606803 PMC2884457

[B6] Okunaga T Izumo T Yoshioka T Shimizu T Yamashita H Yokoyama H A case of transorbital penetrating brain injury by a blunt metal rod No Shinkei Geka 2010 38 3 293 298 20229776

[B7] Pascual JM Navas M Carrasco R Penetrating ballistic-like frontal brain injury caused by a metallic rod Acta Neurochir 2009 151 6 689 691 10.1007/s00701-009-0222-8 19277462

[B8] Regev E Constantini S Pomeranz S Sela M Shalit M Penetrating craniocerebral injury caused by a metal rod: an unusual case report Injury 1990 21 6 414 415 10.1016/0020-1383(90)90138-K 2276815

[B9] Stone JL Rifai MH Moody RA An unusual case of penetrating head injury with excellent recovery Surg Neurol 1981 15 5 369 371 10.1016/0090-3019(81)90173-7 9760976

[B10] Mason F Case of a young man who had a pitchfork driven into his head four inches who speedily got well. Mar 10, 1806 Lancet 1870 1 700 701

[B11] Penfield W Buckley R Punctures of the brain Arch Neurol Psychiat 1928 20 1 13 10.1001/archneurpsyc.1928.02210130004001

[B12] Pilcher C Penetrating wounds of the brain Ann Surg 1936 103 173 198 10.1097/00000658-193602000-00003 17856711 PMC1391014

[B13] Markham JW Mccleve PE Lynge HN Penetrating craniocerebral injuries J Neurosurg 1964 21 1095 1097 10.3171/jns.1964.21.12.1095 14279830

[B14] Dooling JA Bell WE Whitehurst WR Penetrating skull wound with a pair of scissors. Case report J Neurosurg 1967 26 636 638 10.3171/jns.1967.26.6.0636 6027450

[B15] Gulati A Srinivasan B Hunter R Flood TR Penetrating knife injury to the frontal lobe--a case report Ann R Coll Surg Engl 2010 92 6 W41 W42 10.1308/147870810X12699662981672 20615305 PMC5696877

[B16] Mitilian D Charon B Brunelle F Di Rocco F Removal of a chopstick out of the cavernous sinus, pons, and cerebellar vermis through the superior orbital fissure Acta Neurochir 2009 151 10 1295 1297 10.1007/s00701-009-0418-y 19499165

[B17] Schreckinger M Orringer D Thompson BG La Marca F Sagher O Transorbital penetrating injury: case series, review of the literature, and proposed management algorithm J Neurosurg 2011 114 1 53 61 10.3171/2010.8.JNS10301 20868210

[B18] Kim SW Youn SK Kim JT Cho SH Kim YH Hwang KT Management of an unusual craniofacial impalement injury by a metallic foreign body J Craniofac Surg 2012 23 2 e140 e146 10.1097/SCS.0b013e31824cdc2b 22446451

[B19] Iwakura M Kawaguchi T Hosoda K Shibata Y Komatsu H Yanagisawa A Kohmura E Knife blade penetrating stab wound to the brain--case report Neurol Med Chir 2005 45 3 172 175 10.2176/nmc.45.172 15782012

[B20] Chibbaro S Tacconi L Orbito-cranial injuries caused by penetrating non-missile foreign bodies. Experience with eighteen patients Acta Neurochir 2006 148 9 937 941 10.1007/s00701-006-0794-5 16763734

[B21] Lindenberg R Minckler J Trauma of meninges and brain Pathology of the Nervous System. Volume 2 1971 New York: McGraw-Hill 1721

[B22] Khalil N Elwany MN Miller JD Transcranial stab wounds: morbidity and medicolegal awareness Surg Neurol 1991 35 4 294 299 10.1016/0090-3019(91)90008-W 2008646

[B23] De Villiers JC Vinken PJ, Bruyn JW Stab wounds of the brain and skull Handbook of clinical neurology. Vol. 1 1975 Amsterdam: North Holland Ed 477 493

[B24] De Villiers JC Sevel D Intracranial complications of transorbital stab wounds Br J Ophthalmol 1975 59 1 52 56 10.1136/bjo.59.1.52 1125159 PMC1017344

